# Psychological and Physiological Effects of the Mindful Lovingkindness Compassion Program on Highly Self-Critical University Students in South Korea

**DOI:** 10.3389/fpsyg.2020.585743

**Published:** 2020-10-14

**Authors:** Seunghye Noh, Hyunju Cho

**Affiliations:** Department of Psychology, Yeungnam University, Gyeongsan-si, South Korea

**Keywords:** self-criticism, mindfulness, lovingkindness, compassion, HRV

## Abstract

**Objectives:**

Self-critical behavior is especially relevant for university students who face academic and non-academic stressors, leading to negative outcomes such as mental distress and psychopathologies. To address this behavior, mindfulness and compassion are important factors to decrease self-criticism and ensure positive outcomes. This study examined the psychological and physiological effects of an intervention, the Mindful Lovingkindness Compassion Program (MLCP), on highly self-critical university students in South Korea.

**Methods:**

Thirty-eight university students with a high level of self-criticism were assigned to an MLCP group (*n* = 18) or waitlist (WL) group (*n* = 20). Self-report measures of self-criticism, self-reassurance, psychological distress, and other mental health variables were completed, and the physiological measure of heart rate variability (HRV) was conducted before and after the intervention with both groups. In addition, 1- and 3-month follow-up assessments were conducted using self-report measurements.

**Results:**

Compared to the WL group, participants in the MLCP group experienced significantly greater reductions in self-criticism and psychological distress, and a greater increase in self-reassurance, mental health, and HRV. The improvements in the self-report measures were maintained when assessed 1 and 3 months later.

**Conclusions:**

MLCP could be a promising intervention for alleviating self-criticism and increasing self-reassurance among self-critical individuals.

## Introduction

Self-criticism (SC) is a self-evaluative process that involves negative thoughts about various aspects of the self (e.g., appearance, personality, behavior, intelligence, performance, and so forth) (Blatt, 1974; Gilbert and Procter, 2006). Individuals who engage in SC have a punitive stance toward the self when their inner expectations and standards are not met (Blatt and Zuroff, 1992; Cho et al., 2019). On the other hand, SC has an adaptive function, for example, monitoring and correcting one’s mistakes via SC leads to better performance (Gilbert et al., 2004). Nevertheless, excessive SC could be a common risk factor for various forms of mental distress and psychopathologies, including not only depression but also social anxiety (Shahar et al., 2015), eating disorders (Fennig et al., 2008), and post-traumatic stress disorder (Harman and Lee, 2010). In addition, SC can cause negative outcomes in psychotherapy sessions by breaking the therapeutic alliance between the patient and the therapist (Kannan and Levitt, 2013; Werner et al., 2019). A study suggested that the subsyndromal symptom rate among university students was higher than that of the general population, suggesting that they are an at-risk population of experiencing poor mental health (Stallman, 2010). This is because university students experience psychological demand when they adapt to a new social context (Storrie et al., 2010). They experience not only the academic (i.e., poor grades) problems but also non-academic ones, such as socially related stressors (i.e., social isolation) and financial problems. When these new demands of university life interact with their self-criticism, they are at a potentially high risk of experiencing psychological distress (Campos et al., 2018). Therefore, it is necessary to prevent unhealthy SC among university students before this leads to them developing other mental disorders.

There are two pathologic aspects of SC: first, the degree of self-directed contempt that pervades SC is noticeable (Whelton and Greenberg, 2005). While self-critical individuals were easily able to access self-critical images, they lacked the ability to generate self-reassuring images (Gilbert et al., 2006). Therefore, this shows that individuals with high levels of SC are hostile to the self and perceive the self as inadequate and as an object of hatred. Therefore, they treat themselves harshly and apply rigid standards to themselves (Cho et al., 2019). In addition, these self-attacking processes are highly fused with shame that, when associated with SC, increases their vulnerability to a range of difficulties (Gilbert and Irons, 2004, 2009). Second, previous studies have indicated that SC could be a habitual and unconscious response that occurs when self-critical schemas are triggered (Rahamim et al., 2016; Cho et al., 2019). Verplanken et al. (2007) have suggested that the difficulties in controlling self-critical thoughts predict future psychopathology (Verplanken et al., 2007).

Compassion, which is known to be a protective factor for various types of distress and psychopathology (Gilbert, 2000), involves the motivation to be sensitive to suffering and to try to commit to alleviating it (Gilbert, 2014). Compassion-based interventions (e.g., compassion-focused therapy; CFT), which focus on the first feature of SC (self-directed contempt), were effective for highly self-critical individuals (Gilbert and Irons, 2004; Gilbert and Procter, 2006). Furthermore, the CFT significantly decreased SC, shame, and other variables related to mental distress, such as depression, anxiety, and stress. In addition, there were significant increases in self-soothing behaviors and reassuring the self (Gilbert and Procter, 2006). However, CFT may be limited in engaging in the second feature of SC, which is an automatic process of SC. As previously mentioned, SC is a type of mental habit in which people subconsciously blame themselves (Verplanken et al., 2007; Cho et al., 2019). Therefore, being aware of the contents and the process of SC and bringing one’s attention to the here and now are required when people criticize themselves.

Mindfulness is the process of, purposefully and without judging oneself, paying attention to the present moment (Kabat-Zinn, 1994). For example, in mindfulness meditation, people are guided to bring their attention to their present experiences (i.e., breathing, emotions, and thoughts) and to observe these without judgment rather than being overwhelmed by these experiences. To our knowledge, no empirical studies have been conducted to investigate the direct effects of mindfulness meditation on self-critical thoughts. However, a study have shown that people with high levels of trait mindfulness, compared to those with low levels, had low frequencies of negative thoughts (Frewen et al., 2008). Also, studies that have reviewed the benefits of mindfulness-based interventions have found that it reduced stress, anxiety, and other forms of mental distress and improved psychological well-being (see Davis and Hayes, 2011). Furthermore, de-automatizing, a mindfulness meditation mechanism, helped individuals be aware of automatic processes and improved their adaptive self-regulation (Shapiro et al., 2006). Therefore, once individuals become aware of their critical thinking through mindfulness meditation and bringing attention to the here and now, they are most likely to stop this negative behavior.

Studies have also shown that some individuals with high levels of SC resist the compassionate mind (Gilbert et al., 2011). This is because individuals with high levels of SC are afraid of becoming weak; thus, they feel that they have lost the competition with themselves to avoid being weak when they treat themselves with compassion (Gilbert and Choden, 2013). Therefore, if we do not take the fear of compassion into account when addressing SC, individuals will resist the compassionate mind even more. At this point, mindfulness meditation would increase awareness of people’s resistance to compassion. Through mindfulness meditation, individuals may gain an understanding of their fear by recognizing how it arises and how much pain they are experiencing (Kabat-Zinn, 1994; Davis and Hayes, 2011; Gilbert and Choden, 2013).

Overall, two essential factors are required when intervening in highly self-critical behavior of individuals: (1) mindfulness (being aware of self-critical thinking and the fear of compassion) and (2) lovingkindness and compassion (cultivating the ability to be compassionate to oneself and others). Therefore, the Mindful Lovingkindness Compassion Program (MLCP), which was developed for novice counselors and therapists to help their growth and to reduce burnout (Cho et al., 2014), is suitable for highly self-critical individuals as it contains both mindfulness and compassion.

Overall, The MLCP consists of two main parts: (1) mindfulness meditation, which involves noticing the elements of the mind (sensation, emotion, thoughts, and desire) and understanding their connections, and (2) unification of the multiple selves when being compassionate to oneself, extending compassion outwards, and establishing a feeling of connection with the world by practicing compassion (Cho et al., 2014). In total, there are eight sessions in this program: the first three sessions include mindfulness meditation and the later five sessions focus on compassion practices, but all sessions start and finish with mindfulness meditation. The MLCP is different from other compassion-based interventions because it particularly emphasizes the following aspects: first, it highlights seven mindful attitudes and a noble eightfold path during all the sessions (e.g., non-judgment, patience, non-striving); second, the concept of lovingkindness and compassion is not just the combination of two words; instead, it is based on an interdependent origination, which emphasizes the connection with all living things; third, it focuses on the natural flow of compassion by receiving a compassionate mind from an ideal compassionate nurturer (who is created in the person’s imagination), cultivating the compassionate self, unifying the multiple selves, and gradually extending it from those who are close toward oneself to others. Table 1 presents all the sessions of the MLCP. Furthermore, the MLCP has been shown to be effective among university students with high levels of depression (Jeong et al., 2017) and social anxiety disorder (Ryu, 2019).

**TABLE 1:** Entire sessions of the Mindful Lovingkindness Compassion Program.

**Session**	**Theme**	**Summary of the practices**
1	Mindfulness meditation 1	Exploring participants needs and expectations; introducing the MLCP, nature of the mind, self-criticism, and basic attitude when practicing the meditation; practicing the mindfulness meditation (focus on breathing)
2	Mindfulness meditation 2	Introducing the mindful meditation about sound, body sensation; practicing the mindfulness meditation (focus on sound and bodily sensation)
3	Mindfulness meditation 3	Introducing the mindful meditation about the thoughts, emotions, and self-critical events
4	Lovingkindness and compassion practice 1	Introducing the lovingkindness and compassion and emotion regulation system; practicing the lovingkindness and compassion using the imagination (safe place, ideal compassionate nurturer)
5	Lovingkindness and compassion practice 2	Finding one’s compassionate attributes and share them with the group; imaging lovingkindness and compassion to oneself
6	Lovingkindness and compassion practice 3	Meditating lovingkindness and compassion to self-critical self; writing compassionate letter to oneself
7	Lovingkindness and compassion practice 4	Meditating lovingkindness and compassion to loved ones, group members, and strangers
8	Lovingkindness and compassion practice 5	Exploring and discussing the meaning of one’s life; meditating the meaning of one’s life; reviewing the entire session and discussing the application of the practices

*All sessions start with discussing the experience of personal practices and mindful breathing meditation and finish with sharing the experiences of the session.*

The concept of compassion includes motivation, affection, cognition, and behavior that the effects of compassion-based interventions would consider with physiological mechanisms. According to the Polyvagal theory (Porges, 2003), compassion practices soothe our defense (sympathetic nervous) system and promote safeness (parasympathetic nervous). In other words, the vagal nervous system balances the sympathetic and parasympathetic nervous systems that contribute to variability in heart rate and is associated with the ability to regulate emotion and social connectedness (Porges, 2003). Therefore, heart rate variability (HRV) is an essential measure of the physiological effect of compassion and of overall health, with low levels of HRV being linked to mental distress and high levels of HRV being associated with compassion (Kirby et al., 2017a). Although compassion involves a physiological response, most previous studies have relied on subjective measurements when investigating its effects (Kirby et al., 2017a). To the best of our knowledge, few studies have tried to verify compassion’s physiological effects among highly self-critical individuals (Halamová et al., 2019; Rockliff et al., 2008; Rose et al., 2018). One study investigated the differences in HRV with different SC levels during exposure to a guided imagery task (Halamová et al., 2019). It was found that individuals with high levels of SC had low HRV compared to individuals with low levels of SC, and people showed high HRV when they viewed compassionate imagery. Thus, the effects of lovingkindness and compassion need to be verified with HRV, which is associated with emotion regulation and social connectedness.

The present study investigated the psychological and physiological effects of the MLCP on highly self-critical individuals. We hypothesized that (1) individuals who participated in the MLCP (the MLCP group) would exhibit reduced shame, SC, and fear of compassion toward the self and have fewer mental health problems (depression, anxiety, and stress) compared to the waitlist (WL) group who waited 6 weeks before participating in the intervention; (2) the MLCP group would improve in terms of mindfulness, self-reassurance, compassion, and life satisfaction compared to the WL group; (3) the MLCP group would experience changes in HRV, whereas the WL group would not; and (4) there would be lasting effects for the MLCP group. This study is one of the first to examine the effects of the MLCP on self-critical individuals using both psychological and physiological assessments.

## Materials and Methods

### Participants

Forty students with high levels of SC participated in this study. The sample of university students in South Korea was recruited both offline and online. A total of 463 students completed the Forms of Self-Criticizing/Attacking (SC) and Self-Reassuring Scale (FSCRS; Gilbert et al., 2004) to ensure that the sample was highly self-critical. Seventy students were interested in taking part in the study, but 30 students were excluded due to not meeting the inclusion criteria. We defined a high level of SC as a score of over 23 points on the SC subscale and under 21 points on the Self-reassuring (SR) subscale (Baião et al., 2015). Thus, the exclusion criteria were (a) obtaining a score of less than 23 on the SC subscale and more than 21 on the SR subscale of the FSCRS, (b) undergoing concurrent psychotherapy for self-criticism, and (c) taking any psychotropic medication. We assigned several participants who want to participate in the program as soon as possible to the MLCP group, and then the rest to the MLCP group and WL group according to the order they arrived in the laboratory for the measuring baseline. Therefore, *n* = 20 students attended at least one MLCP session, *n* = 2 (10%) MLCP dropped out after the first and third session, and *n* = 3 (16.6%) MLCP did not complete the 3-month follow-up assessment. Finally, *n* = 18 (90%) MLCP took part in the pre-, post-, and follow-up assessment 1 month after the intervention and *n* = 15 (75%) MLCP took part in the follow-up assessment 3 months after the intervention. There was no dropout in the WL group; therefore, *n* = 20 WL completed the post-assessment after the intervention.

The mean age of the participants was 21.50 years [standard deviation (*SD*) = 1.69] in the MLCP group and 21.60 (*SD* = 2.28) in the WL group. Regarding the gender, 66.70% were female (*n* = 12) in the MLCP group and 50% were female (*n* = 10) in the WL group. There were no significant differences between the groups in terms of gender, χ^2^(1, *n* = 38) = 1.10, *p* = 0.30, and age, *t*(36) = −0.15, *p* = 0.88.

### Procedure

All participants were invited to the laboratory before starting the program and were informed about the procedure of the study. All participants provided informed consent and completed the psychological assessment (i.e., the self-report measures) and then the physiological assessment. Participants were asked to refrain from (a) eating; (b) drinking alcohol, tea, or coffee; and (c) performing strenuous exercise for 2–3 h preceding the scheduled appointment. After completing the self-report measures (time 1), the participants were asked to lie down and to relax for 5 min in order to obtain the measures of the resting-state HRV. Both groups underwent post-intervention assessments (time 2), which were the same as time 1. Participants in the MLCP group were also asked to answer the self-report measures after 1 month (time 3) and 3 months (time 4) had elapsed to evaluate the prolonged effects of the MLCP.

Participants in the MLCP group took part in eight sessions over the course of 6 weeks and with each session lasting 100–120 min. They were provided with files of the MLCP practice for individual practice, while the WL group waited for 6 weeks. Each session consisted of sharing their personal training, guiding the training of each daily session, practicing them and sharing their experience. The WL group was offered the opportunity to attend MLCP after the waiting period. All participants received compensation for their participation (20,000 KRW). The study procedures were reviewed and approved by IRB (Institutional Review of Yeungnam University; YU 2018-08-001-001).

### Instructors

The first author instructed the MLCP. She was a certified professional teacher in meditation—temporarily a member of the Korean Society for Meditation, had been trained by Ph.D. Cho (who was a licensed clinical psychologist and certified professional teacher in meditation registered on the Korean Society for Meditation), and had 6 years of experience as a meditation instructor.

### Measures

#### FSCRS

The FSCRS was developed by Gilbert et al. (2004) to measure the forms of people’s critical and reassuring self-evaluative responses when things go wrong for them. The original scale consisted of 22 items. An 18-item scale, which was validated with Korean university students, was used in this study (Cho, 2011). The FSCRS consisted of two factors; SC (self-criticism) includes 10 items and the SR (self-reassurance) includes 8 items. Participants responded on a five-point Likert scale (ranging from 0 = “not at all like me” to 4 = “extremely like me”). Consistency values for the FSCRS were α = 0.85–88 in the current sample.

#### State Mindfulness Scale (SMS)

The SMS was developed to measure the state of mindfulness (Tanay and Bernstein, 2013). It was designed to assess mindfulness during mindfulness practice and reflect the traditional Buddhist and contemporary mindfulness model. The Korean version of SMS (Noh et al., 2019) consists of 21 and 2 factors: state mindfulness of the mind and state mindfulness of the body. The participants were asked to respond to each item based on their experience in the past 15 min on a five-point Likert scale (ranging from 1 = “not at all” to 5 = “very well”). Consistency values for the SMS were α = 0.93–0.94 in the current sample.

#### Loving-Kindness Compassion Scale (LCS)

The LCS was developed to measure lovingkindness and compassion based on the Buddhist tradition (Cho et al., 2018). It highlights the boundless state of the mind; that is, that all living beings are willing to be released from suffering and to be happy. The LCS is composed of 15 items, and it has three subscales: lovingkindness, compassion, and self-centredness. Participants respond on a five-point Likert scale (ranging from 1 = “not at all true of me” to 5 = “very true of me”). Consistency values for the LCS were α = 0.72–0.85 in the current sample.

#### Internalized Shame Scale (ISS)

The ISS is a 30-item self-report scale that is designed to measure trait shame, and it consists of two subscales: a 24-item subscale that assesses trait shame and a 6-item subscale that measures self-esteem (Cook, 2001). The latter items are adapted from Rosenberg’s Self-Esteem Scale (Rosenberg, 1965) and they are used in the ISS to prevent response set bias. Participants rate each item on a five-point Likert scale (ranging from 0 = “never” to 4 = “almost always”), which was validated with Korean students (Lee and Choi, 2005). The six self-esteem items were not included when the total shame score was calculated. Consistency values for the ISS were α = 0.88–0.92 in the current sample.

#### Depression Anxiety Stress Scales 21 (DASS 21)

The Korean version of DASS 21 (Psychology Foundation of Australia, 2013) is used to measure the participants’ level of psychological distress. It is composed of 21 items, and it has three subscales: depression, anxiety, and stress. Participants are required to respond based on their experience during the previous week. They rate each item on a four-point Likert scale (ranging from 0 = “does not apply to me at all” to 3 = “applies to me very much, or most of the time”). Consistency values for the DASS 21 were α = 0.91–0.93 in the current sample.

#### Fears of Compassion Scale-Self (FC-Self)

Gilbert et al. (2011) developed the Fears of Compassion Scale. It includes three domains of fears of compassion: fears and difficulties in receiving compassion from others, in expressing compassion for others, and in compassion for the self. In this study, only “for the self” was used because individuals with a high level of SC are afraid of feeling compassion for themselves. The FC-Self, validated with Korean students (Joeng et al., 2015), is composed of 15 items (e.g., “I feel that I do not deserve to be kind and forgiving to myself”). Participants respond on a five-point Likert scale (ranging from 1 = “do not agree at all” to 5 = “completely agree”). Consistency values for the FC-Self were α = 0.83–0.91 in the current sample.

#### Satisfaction With Life Scale (SWLS)

The SWLS (Diener et al., 1985) was used to measure the degree of overall life satisfaction. It was validated with Korean population (Lim, 2012) and composed of five items. Participants respond on a seven-point Likert scale (ranging from 1 = “not at all” to 7 = “very well”). Consistency values for the SWLS were α = 0.82–0.90 in the current sample.

#### HRV

The HRV-related components were obtained using the STD-1000k system (StraTek Co., Anyang, Korea). The device was attached to participants using a four-lead electrocardiogram connector on both wrists and ankles. The time domain of the HRV included the standard deviation of all normal-to-normal intervals (SDNN) and the frequency domain of the HRV included high frequency (HF). The domains are known to reflect the adaptive physiological regulation ability; SDNN is recommended as a global measure of respiratory-linked variability (Allen et al., 2007) and HF HRV relates to parasympathetic activation (Thayer and Lane, 2007). All data were saved automatically in the STD-1000k program. A lower value of the index indicates a higher level of stress.

### Data Analysis

The data were analyzed using SPSS Statistics for Windows, version 25.0 (IBM Corp., Armonk, NY, United States) and checked for the normality of distribution. A visual inspection of histograms, normal *Q*–*Q* plots, and box plots showed that the measures were approximately normally distributed between groups, with a skewness range from -0.60 to 1.21 and a kurtosis range from -1.62 to 1.41 for the MLCP group and -0.46 to 1.70 and -1.00 to 3.22 for the WL group, respectively (Kline, 2005). A Levene’s test verified the equality of variances in the sample (homogeneity of variance) (*p* > 0.05) (Martin and Bridgmon, 2012).

Independent *t-*tests were conducted to analyze differences between the MLCP and WL at baseline. To investigate the effectiveness of the MLCP on participants’ level of all psychological measures, 2 (Group: MLCP, WL) × 2 (Time: time 1, time 2) repeated-measures MANOVA was performed. The analysis revealed significant interaction effect, Wilks’ Δ = 0.40, *F*(7, 252) = 17.29, *p* < 0.001, η*_*p*_*^2^ = 0.32. We also investigated the effectiveness of the MLCP on participants’ level of all physiological measures; 2 (Group: MLCP, WL) × 2 (Time: time 1, time 2) repeated-measures MANOVA was performed. The analysis revealed significant effect, Wilks’ Δ = 0.87, *F*(1, 29) = 4.37, *p* < 0.05, η*_*p*_*^2^ = 0.13. Then, the study employed a repeated-measures ANOVA design of 2 (Group: MLCP, WL) × 2 (Time: time 1, time 2) investigating different effects between groups for each dependent variable. Where significant interaction effects were found, *post hoc* analysis were conducted using paired *t-*tests. Partial η^2^ was calculated as estimates of effect sizes (Richardson, 2011). According to Cohen (1992), a partial η^2^ of 0.01 indicates a small effect size, 0.06 indicates a medium effect size, and 0.14 indicates a large effect size. The effect sizes for the paired sample *t-*tests were calculated using Cohen *d*, with 0.20 indicating a small effect, 0.50 indicating a medium effect, and 0.80 indicating a large effect size (Cohen, 1992). All the measurements were conducted based on (a) program completers and (b) the intent-to-treat (ITT) sample. For the ITT analysis, we used a standard conservative method in which the participant’s last observation was carried forward to account for missing data. Because the completer and ITT analyses yielded largely similar results, here we reported only the completer analyses (results of the ITT analyses are available upon request).

Lastly, a within-subjects one-way ANOVA was performed to examine if the effect of the MLCP was maintained from the post-intervention assessment to the 1- and 3-month follow-up.

## Results

The MLCP and WL groups did not differ in any of the variables of the self-report measures or the physiological measurement at baseline (all *p*s > 0.05). The specific results were as follows. SC, *t*(36) = 0.89, *p* = 0.38; SR, *t*(36) = -0.76, *p* = 0.45; SMS, *t*(36) = -1.69, *p* = 0.10; LCS, *t*(36) = -1.30, *p* = 0.20; ISS, *t*(36) = 1.35, *p* = 0.19; DASS 21, *t*(36) = 1.77, *p* = 0.09; FC-Self, *t*(36) = 0.02, *p* = 0.99; and SWLS, *t*(36) = -1.13, *p* = 0.27. Furthermore, the groups did not differ on SDNN, *t*(29) = 1.21, *p* = 0.24, and HF, *t*(29) = 1.24, *p* = 0.23 (see Tables 2, 4 for means, standard deviations, and statistics).

**TABLE 2:** Repeated-measures analysis of variance (psychological measurement).

**Variable**	**MLCP group (*n* = 18)**			**WL group (*n* = 20)**			**Group**	**Time**	**Group × Time**
			
	***M* (*SD*)**			***M* (*SD*)**			***F* (η*_*p*_*^2^)**		
	**T1**	**T2**	***d*^1^**	**T1**	**T2**	***d*^2^**			
SC	27.94 (3.46)	17.06 (4.70)	2.64	26.85 (4.04)	23.85 (5.20)	0.64	6.26*(0.15)	63.52***(0.64)	20.49***(0.36)
SR	14.11 (4.32)	21.72 (3.00)	2.05	15.05 (3.22)	16.55 (3.56)	0.44	4.34*(0.11)	70.55***(0.66)	31.74***(0.47)
SMS	48.06 (14.89)	70.72 (16.73)	1.43	56.50 (15.87)	57.60 (15.66)	0.07	0.27 (0.01)	22.34***(0.38)	18.40***(0.34)
LCS	41.89 (7.16)	56.50 (7.40)	2.00	44.75 (6.37)	49.90 (8.77)	0.67	1.02 (0.03)	38.84***(0.52)	8.90**(0.20)
ISS	46.56 (18.58)	24.94 (12.93)	1.35	38.90 (16.52)	39.30 (24.04)	0.02	0.38 (0.01)	16.10***(0.31)	17.34***(0.33)
DASS 21	23.22 (11.85)	11.28 (10.25)	1.08	16.75 (10.73)	17.95 (11.87)	0.11	0.00 (0.00)	7.95**(0.18)	11.80**(0.25)
FC-S	42.00 (9.91)	30.17 (8.52)	1.28	41.95 (8.96)	38.58 (9.90)	0.36	2.73 (0.07)	20.97***(0.37)	6.43*(0.15)
SWLS	15.61 (4.68)	20.83 (4.83)	1.10	17.25 (4.27)	18.05 (4.43)	0.18	0.18 (0.01)	26.99***(0.43)	14.55**(0.29)

*MLCP, Mindfulness Lovingkindness Compassion Program; WL, waitlist; d^1^*, *Cohen’s d of the MLCP (T1 - T2); d^2^*, Cohen’s d of the WL (T1 - T2); SC, self-criticism; SR, self-reassuring; SMS, State Mindfulness Scale; LCS, Lovingkindness Compassion Scale; ISS, Internalized Shame Scale; DASS 21, Depression Anxiety Stress Scales; FC-S, Fears of Compassion Scale-Self; SWLS, Satisfaction With Life Scale; T1, pre-intervention; T2, post-intervention. * p < 0.05, ** p < 0.01, *** *p < 0.001.*

### The Psychological Effects of the MLCP

A 2 (Group: MLCP, WL) × 2 (Time: time 1, time 2) repeated-measures ANOVA was conducted for all the variables. The main results are presented in Table 2. For SC, the effects of Group, *F*(1, 36) = 6.26, *p* < 0.05, η*_*p*_*^2^ = 0.15, and Time, *F*(1, 36) = 63.52, *p* < 0.001, η*_*p*_*^2^ = 0.64, were significant. The results indicated that the total SC scores were lower in the MLCP group compared to the WL group and decreased over time. The interaction of Group × Time, *F*(1, 36) = 20.49, *p* < 0.001, η*_*p*_*^2^ = 0.36, was also significant. *Post hoc* analysis revealed that participants in the MLCP group had significantly lower time 2 total SC scores, *t*(17) = 7.46, *p* < 0.001, *d* = 2.64. Participant in the WL group also had lower time 2 total SC scores, *t*(19) = 2.98, *p* < 0.01, *d* = 0.64.

For SR, there were significant effects of Group, *F*(1, 36) = 4.34, *p* < 0.05, η*_*p*_*^2^ = 0.11, and significant effect of Time, *F*(1, 36) = 70.55, *p* < 0.001, η*_*p*_*^2^ = 0.66. The results indicated that the total SR scores were higher in the MLCP group compared to the WL group and increased over time. The interaction of Group × Time, *F*(1, 36) = 31.74, *p* < 0.001, η*_*p*_*^2^ = 0.47, was also significant. *Post hoc* analysis revealed that participants in the MLCP group had higher time 2 total SR scores, *t*(17) = -8.89, *p* < 0.001, *d* = 2.05. Participants in the WL group also had lower time 2 total SR scores, *t*(19) = -2.20, *p* < 0.05, *d* = 0.44.

For the SMS, there was no effect of Group, *F*(1, 36) = 0.27, *p* = 0.60, η*_*p*_*^2^ = 0.01, but the effect of Time, *F*(1, 36) = 22.34, *p* < 0.001, η*_*p*_*^2^ = 0.38, was significant. The results indicated that the total SMS scores increased over time. The Group × Time interaction was significant, *F*(1, 36) = 18.40, *p* < 0.001, η*_*p*_*^2^ = 0.34. *Post hoc* analysis revealed that participants in the MLCP group had higher time 2 total SMS scores, *t*(17) = -5.17, *p* < 0.001, *d* = 1.43. No significant change was found in SMS scores in the WL group, *t*(19) = -0.41, *p* = 0.69, *d* = 0.07.

For the LCS, there was no effect of Group, *F*(1, 36) = 1.02, *p* = 0.32, η*_*p*_*^2^ = 0.03; however, the effect of Time, *F*(1, 36) = 38.84, *p* < 0.001, η*_*p*_*^2^ = 0.52, was significant. The results indicated that the total LCS scores increased over time. The Group × Time interaction was significant, *F*(1, 36) = 8.90, *p* < 0.01, η*_*p*_*^2^ = 0.20. *Post hoc* analysis revealed that the MLCP group had higher time 2 total LCS scores, *t*(17) = -7.96, *p* < 0.001, *d* = 2.00. Participants in the WL group tend to have lower time 2 total SR scores, *t*(19) = -2.05, *p* = 0.05, *d* = 0.67.

For the ISS, there was no effect of Group, *F*(1, 36) = 0.38, *p* = 0.54, η*_*p*_*^2^ = 0.01, whereas there was significant effect of Time, *F*(1, 36) = 16.10, *p* < 0.001, η*_*p*_^2^* = 0.31. The results indicated that the total ISS scores decreased over time. The Group × Time interaction was significant, *F*(1, 36) = 17.34, *p* < 0.001, η*_*p*_*^2^ = 0.33. *Post hoc* analysis revealed that participants in the MLCP group had higher time 2 total ISS scores, *t*(17) = 6.09, *p* < 0.001, *d* = 1.35. No significant change was found in ISS scores in WL group, *t*(19) = -0.10, *p* = 0.92, *d* = 0.02.

For the DASS 21, there was no effect of Group, *F*(1, 36) = 0.00, *p* = 0.97, η*_*p*_*^2^ = 0.00, but there was significant effect of Time, *F*(1, 36) = 7.95, *p* < 0.01, η*_*p*_*^2^ = 0.18. The results indicated that the total DASS 21 decreased over time. The Group × Time interaction was significant, *F*(1, 36) = 11.80, *p* < 0.01, η*_*p*_*^2^ = 0.25. *Post hoc* analysis revealed that the MLCP group had lower time 2 total DASS 21 scores, *t*(17) = 3.49, *p* < 0.01, *d* = 1.08. No significant change was found in DASS 21 scores in the WL group, *t*(19) = -0.63, *p* = 0.53, *d* = 0.11.

For the FC-Self, there was no effect of Group *F*(1, 36) = 2.73, *p* = 0.11, η*_*p*_*^2^ = 0.07; however, the Time effect was significant, *F*(1, 36) = 20.97, *p* < 0.001, η*_*p*_*^2^ = 0.37. The result indicated that the total FC-Self decreased over time. The Group × Time interaction was significant, *F*(1, 36) = 6.43, *p* < 0.05, η*_*p*_*^2^ = 0.15. *Post hoc* analysis revealed that the MLCP group had lower time 2 total FC-Self scores, *t*(17) = 4.10, *p* < 0.01, *d* = 1.28. No significant change was found in FC-Self scores in the WL group, *t*(19) = 1.89, *p* = 0.07, *d* = 0.36.

For the SWLS, there was no effect of Group *F*(1, 36) = 0.18, *p* = 0.68, η*_*p*_*^2^ = 0.01, but the Time effect was significant, *F*(1, 36) = 26.99, *p* < 0.001, η*_*p*_*^2^ = 0.43. The result indicated that the total SWLS increased over time. The Group × Time interaction effect was significant, *F*(1, 36) = 14.55, *p* < 0.01, η*_*p*_*^2^ = 0.29. *Post hoc* analysis revealed that the MLCP group had higher time 2 total SWLS scores, *t*(17) = -4.92, *p* < 0.001, *d* = 1.10. No significant change was found in SWLS scores in the WL group, *t*(19) = -1.47, *p* = 0.16, *d* = 0.18.

All variables had large interaction effect sizes with partial eta squares ranging from 0.15 to 0.47. The MLCP group had large effect sizes with all variables, whereas the WL group showed medium effect sizes with SC and LCS, small effect sizes with SR and FC-S, and no effect sizes with other variables including SMS, ISS, DASS 21, and SWLS.

### The Lasting Psychological Effects of the MLCP

In addition, a significant effect of Time (T2, T3, T4) was not found for all the variables, which indicated that the effects of the intervention were maintained for 1 and 3 months in the MLCP group, SC, *F*(2, 28) = 1.07, *p* = 0.36, η*_*p*_*^2^ = 0.07; SR, *F*(2, 28) = 1.57, *p* = 0.23, η*_*p*_*^2^ = 0.10; SMS, *F*(2, 28) = 2.13, *p* = 0.14, η*_*p*_*^2^ = 0.13; LCS, *F*(2, 28) = 2.89, *p* = 0.07, η*_*p*_*^2^ = 0.17; ISS, *F*(2, 28) = 0.82, *p* = 0.45, η*_*p*_*^2^ = 0.06; DASS 21, *F*(2, 28) = 0.47, *p* = 0.63, η*_*p*_*^2^ = 0.03; FC-S, *F*(2, 28) = 0.25, *p* = 0.78, η*_*p*_*^2^ = 0.02; SWLS, *F*(2, 28) = 1.22, *p* = 0.31, η*_*p*_*^2^ = 0.08. The results are presented in Table 3.

**TABLE 3:** One-way analysis of variance of T2, T3, and T4 (psychological measurement).

**Variable**	**MLCP group (*n* = 18)**			
	***M* (*SD*)**			
	**T2**	**T3**	**T4^a^**	***F***
SC	17.06 (4.70)	17.78 (6.06)	18.73 (6.08)	1.07
SR	21.72 (3.00)	20.78 (3.57)	22.47 (3.58)	1.57
SMS	70.72 (16.73)	66.00 (17.72)	65.73 (18.39)	2.13
LCS	56.50 (7.40)	52.94 (9.07)	56.27 (7.80)	2.89
ISS	24.94 (12.93)	29.28 (15.01)	26.80 (15.16)	0.82
DASS 21	11.28 (10.25)	13.83 (8.78)	14.67 (9.59)	0.47
FC-S	30.17 (8.52)	29.83 (10.45)	28.07 (10.26)	0.25
SWLS	20.83 (4.83)	18.89 (4.83)	20.60 (4.24)	1.22

*MLCP, Mindfulness Lovingkindness Compassion Program; WL, waitlist; SC, self-criticism; SR, self-reassuring; SMS, State Mindfulness Scale; LCS, Lovingkindness Compassion Scale; ISS, Internalized Shame Scale; DASS 21, Depression Anxiety Stress Scales; FC-S, Fears of Compassion Scale-Self; SWLS, Satisfaction With Life Scale. ^*a*^Three participants’ data were excluded for not receiving the T4.*

### The Physiological Effects of the MLCP

A 2 (Group: MLCP, WL) × 2 (Time: T1, T2) repeated-measures ANOVA was conducted for the physiological variables. The results are presented in Table 4. For the SDNN, the effect of Group, *F*(1, 29) = 6.68, *p* < 0.05, η*_*p*_*^2^ = 0.19, was significant, but the Time effect, *F*(1, 29) = 2.14, *p* = 0.15, η*_*p*_*^2^ = 0.07, was not. This result indicated that the total SDNN scores were higher in the MLCP group compared to the WL group. The Group × Time interaction, *F*(1, 29) = 4.50, *p* < 0.05, η*_*p*_*^2^ = 0.13, was significant. *Post hoc* analysis revealed that the MLCP group had higher time 2 total SDNN scores, *t*(14) = -2.36, *p* < 0.05, *d* = 0.73. No significant change was found in SDNN scores in the WL group, *t*(15) = 0.50, *p* = 0.62, *d* = 0.09.

**TABLE 4:** Repeated-measures analysis of variance (physiological measurement).

**Variable**	**MLCP group (*n* = 15)^*a*^**			**WL group (*n* = 16)^*a*^**			**Group**	**Time**	**Group × Time**
		
	***M* (*SD*)**								
	**T1**	**T2**	***d*^1^**	**T1**	**T2**	***d*^2^**	***F* (η*_*p*_*^2^)**		
SDNN	42.33 (6.75)	46.88 (5.68)	0.73	38.65 (9.74)	37.81 (7.92)	0.09	6.68* (0.19)	2.14 (0.07)	4.50* (0.13)
HF	5.03 (0.48)	5.27 (0.25)	0.63	4.82 (0.47)	4.80 (0.45)	0.04	6.29* (0.18)	2.49 (0.08)	3.40^†^ (0.11)

*MLCP, Mindfulness Lovingkindness Compassion Program; WL, waitlist; T1, pre-intervention; T2, post-intervention; d^1^, *Cohen’s d of the MLCP (T1 - T2); d^2^*, *Cohen’s d of the WL (T1 - T2); SDNN, standard deviation of all normal-to-normal intervals; HF, high frequency heart rate variability.*^*a*^The analyses of the heart rate variability (HRV) included 31 participants. Three participants in the MLCP group and four participants in the WL group were excluded from the analysis of the physiological measurements for the following reasons: in the MLCP group, one did not agree with the measurement of the HRV, and two had slept for less than 4 h the previous night, and in the WL group, one had heart disease and three moved and slept during the HRV measurement. ^∗^*p* < 0.05, ^†^*p* = 0.075.*

For the HF, the effect of Group, *F*(1, 29) = 6.29, *p* < 0.05, η*_*p*_*^2^ = 0.18, was significant, whereas the effect of Time, *F*(1, 29) = 2.49, *p* = 0.13, η*_*p*_*^2^ = 0.08, was not significant. This result indicated that the total HF scores were higher in the MLCP group compared to the WL group. There was a tendency toward a significant Group × Time interaction, *F*(1, 29) = 3.40, *p* = 0.075, η*_*p*_*^2^ = 0.11. *Post hoc* analysis revealed that the MLCP group had higher time 2 total HF scores, *t*(14) = -2.84, *p* < 0.05, *d* = 0.63. No significant change was found in HF scores in the WL group, *t*(15) = 0.17, *p* = 0.87, *d* = 0.04.

The SDNN and HF had medium interaction effect sizes with partial eta squares ranging from 0.11 to 0.13. The MLCP group had large effect sizes with all variables, whereas the WL group showed no effect sizes.

## Discussion

This study examined the psychological and physiological effects of the MLCP on individuals with high levels of SC and compared them to the WL group. We hypothesized that participants receiving the MLCP would show: (1) a decrease in shame, SC, fear of compassion toward the self, depression, anxiety, and stress, as well as (2) an increase in self-reassurance, mindfulness, compassion, and life satisfaction. In addition, (3) these changes would be found in the physiological index, HRV. Lastly, (4) the changes measured by self-report would last at 1 and 3 months follow-up.

The results supported the first and second hypotheses. The MLCP significantly reduced individuals’ SC, shame, distress, and fear of compassion, whereas it significantly improved their SR, compassion, mindfulness, and satisfaction with life compared to the WL group. The significant findings for all variables in the MLCP group were accompanied by large effect sizes. Although differences between pre- and post-intervention were significant on the SC, SR, LCS, and FC-S for both groups, a notable difference in effect size was observed, with the MLCP group displaying large effect sizes (*d* = 1.28–2.64). Findings of the current study are consistent with previous studies that show that MLCP is effective for people who suffer from psychological distress and the number of studies that have shown that mindfulness- or compassion-based programs are effective at enhancing self-soothing and at reducing self-criticism, depression, anxiety, and stress (Gilbert and Procter, 2006; Gu et al., 2015; Arimitsu, 2016; Matos et al., 2017; Rose et al., 2018). The current study suggests that cultivating mindfulness and compassion may be protective interventions when treating high SC individuals.

A question to consider is how does participating in the MLCP lead to these effects? One possible explanation can be the decentering mechanism of mindfulness meditation (Shapiro et al., 2006). In mindfulness meditation, participants are guided to be aware of the moments’ experiences without judgment, and this may prevent people from interminably judging their failures or mistakes, being consumed by negative emotions, and experiencing fear of compassion. Thus, mindfulness meditation enables people to make the choice when habitually engaging in SC to either keep blaming themselves or to stop.

A second possibility for the change is lovingkindness and compassion practices whereby a meta-study supports the idea that compassion-based interventions had a moderate effect size for psychological well-being (see Kirby et al., 2017b). In the compassion practice, the aspect of feeling the ideal compassionate nurturer’s kindness and understanding enables people to find their compassionate self in their mind, enabling them to extend their compassionate self toward the self and others. This result is consistent with prior studies that found that imagining a compassionate mind improved the ability to soothe oneself (Gilbert and Irons, 2004). One participant of the MLCP reported that “I communicated with an anxious self and sad self, which I had hidden.” These empathetic experiences with oneself might be the foundation of extending compassion from oneself toward others in the world. Furthermore, the level of lovingkindness and compassion, which was measured by the LCS, also increased. These results indicate that the participants changed their self-to-self relationship from self-attacking to self-caring and that this caring system was extended toward others. In addition, satisfaction with life increased in the MLCP group, which is consistent with previous studies whereby compassion-based interventions had a moderate effect size for psychological well-being (see Kirby et al., 2017b). Therefore, warm and accepted feelings toward the self and others may activate the soothing system, leading to feelings of safety and kindness toward oneself and others, and overall increased perceived satisfaction with life.

The fear of compassion for the self was significantly decreased in the MLCP group compared to the WL group. This is in line with previous studies that found that compassion-based interventions reduced fear of compassion (Jazaieri et al., 2013; Matos et al., 2017). As it disturbs the self-critical individuals’ access to feeling safe (Gilbert and Irons, 2005; Matos et al., 2017), we guided the fear of compassion from the fourth session, in which the compassionate practice started and lasted for the entire session. Whenever participants were afraid of compassion, we asked them to notice what the fear was. For example, a participant in the MLCP group said that “If I am compassionate to myself, I might be lazy and might not obtain good grades.” We reframed this misunderstanding of compassion and guided the participant to approach his/her concern with kindness. In addition, mindfulness meditation may play a critical role in the person noticing the fear of compassion, therefore leading to them not avoiding the fear but tolerating it. Contrastingly, without mindfulness meditation, they may avoid the fear of compassion, which may be connected to the dropout rate. Therefore, this suggests that the fusion of mindfulness meditation and compassion practice may be effective when applying compassion interventions to self-critical individuals.

The SDNN, which is an indicator of HRV, significantly increased, and the scores of the HF increased, which supported our third hypothesis. Especially, SDNN was marginally associated with increased HF, which is related to positive emotion and connectedness with others and affects regulation (Sahdra et al., 2015). This is consistent with the Polyvagal theory’s proposal that contemplative practice, such as mindfulness and compassion, can calm neural defense systems and promote safe feelings that facilitate social engagement (Khoury, 2019). Our study supports previous research that found that compassion-related interventions increased HRV (Sahdra et al., 2015; Matos et al., 2017; Halamová et al., 2019). Interestingly, the significant improvements in the SDNN and HF scores of the HRV in the MLCP group were associated with large effect sizes (*d* = 0.63–0.73), whereas there were no changes in WL, which is unlike where observed in psychological measurements. These results suggest that physiological measurement could be a critical index to control the participants’ expected effects. The overall findings indicate that the MLCP can help to improve activation of HRV, which relates to safety and relaxation and psychological assessments, however, we should be careful in interpreting the results of the association between compassion and HRV because some studies have shown mixed results for their relationship (Rockliff et al., 2008; Arch et al., 2014; Kirby et al., 2017a) and the measuring points and measurement time vary (e.g., measuring during rest time, a brief intervention/measure for 5 min or 10 min, and so forth).

Finally, the effects of the MLCP measured by self-reports were maintained at 1- and 3-month follow-up, supporting our fourth hypothesis. This result was consistent with the finding in the compassion imagery study (McEwan and Gilbert, 2016) indicating that compassion-based interventions may be effective in protecting against the development of mental disorders. This lasting effect is due to the program’s focus on developing soothing and safe feelings for oneself rather than a logical change in critical thinking with positive affection being developed and expanded as the practice continues. In addition, experiencing a difficult emotion is essential in the MLCP. Furthermore, this is line with the suggestion of Fredrickson et al. (2008) broaden-and-build theory of positive emotions. However, we did not measure positive emotions directly; this will need to be focused on future studies to investigate the mechanisms of effectiveness. In experiencing the MLCP, participants may realize that the difficult emotion will not last forever as they develop the observation of the self through mindfulness meditation. Consequently, this change possibly relates to the lasting effects of the MLCP. In conclusion, our findings highlight how the MLCP is a promising treatment for preventing psychopathology and promoting further well-being among highly self-critical students and further emphasize the benefits of using the MLCP for this purpose. Furthermore, these findings will guide the development of future intervention targeted at addressing SC.

## Limitations and Future Research

There were several limitations in this study. First, while there were no significant differences found in any of the measurements between the groups at pre-intervention, the present study did not employ a randomized controlled study design. Therefore, it did not eliminate potential biases that could have influenced the results and can usually be controlled by randomization. For example, SC, SR, LCS, and FC-S were improved with small to medium effects in the WL group at time 2, however, there were no changes with SDNN and HF in the WL group. These results indicate that it might be due to heightened expectations toward the intervention of treatment. Therefore, this study should be replicated by utilizing a randomized study design to confirm these results.

Second, the sample was a small sample of non-clinical participants from one country, which limited the generalization of our findings. Though the main findings and the effect sizes were reassuring, these findings should be interpreted with caution. Therefore, future studies should replicate these effects with larger samples and clinical samples.

Lastly, we measured HRV, a physiological index, during a rest period. It would be worthwhile to measure the HRV at various time points and to compare them; for example, the HRV could be lower during early practice, even lower when the fear of compassion is aroused, and be higher when the soothing system is activated. Future studies should address this limitation to enable a comprehensive set of findings in this type of sample.

## Data Availability Statement

All datasets presented in this study are included in the article/Supplementary Material.

## Ethics Statement

The studies involving human participants were reviewed and approved by the Yeungnam University’s Institutional Review Board. The patients/participants provided their written informed consent to participate in this study.

## Author Contributions

SN and HC performed the material preparation, data collection, and data analyses. SN wrote the first draft of the manuscript. HC guided and supervised the study and data analyses, and edited the manuscript. Both authors contributed to the study conception and design, read and approved the final manuscript.

## Conflict of Interest

The authors declare that the research was conducted in the absence of any commercial or financial relationships that could be construed as a potential conflict of interest.
