# Supplementary material for: Psychological and Physiological Effects of the Mindful Lovingkindness Compassion Program on Highly Self-Critical University Students in South Korea
Source: Front Psychol. 2020 Oct 14;11:585743. doi: 10.3389/fpsyg.2020.585743 (PMC7591461; doi:10.3389/fpsyg.2020.585743)
Supplement: Supplementary file 2 [file Data_Sheet_2.PDF]

Supplementary Material

1 Supplementary Data

DATA01(PP) Data of pre-intervention and post-intervention (program completers)

DATA01(ITT). Data of pre-intervention and post-intervention (Intent to treat)

DATA02. Data of HRV (pre- and post- intervention)

DATA03. Data of 1- and 3- month follow up
